# Current News and Opinion

**Published:** 1885-01

**Authors:** 


### Current News and Opinion.

### LISTERINE.

As a deodorant and antiseptic for the sick-room and dentist's office, Listerine stands pre-eminent. While it is equal to any and superior to most of the agents commonly used under such circumstances, it adds an agreeable aroma instead of an offensive odor to the surroundings, and is particularly well adapted to the lying-in room. It may be freely used in spray or lotion, without stain or irritation, as an agreeable and effectual detergent. It is also specially commendable in weak solution as a mouth-wash and gargle for aphthous sores or a fungus condition of the gums, and bad breath; and for certain forms of indigestion—those accompanied by disagreeable eructations—a few drops of Listerine in water, swallowed, is a particularly grateful and excellent remedy. Moreover, according to a series of " Experiments upon the Strength of Antiseptics," by Dr. A. T. Cabot (Boston *Medical and Surgical Journal,* Nov. 27, 1879), Listerine compares favorably with the most reliable agents for the rapid destruction of micro-organisms.—*The Sanitarian,* Oct., 1884.

This is " praise from Sir Hubert." Certainly there is no higher authority in America upon antiseptics and general hygiene than is this most excellent journal.

### ANNALS OF SURGERY.

With the new year, J. H. Chambers & Co., the medical publishers of St. Louis, commence the issue of an important journal under the above title. Since the suspension of the *Annals of Anatomy* there has, until now, been nothing to quite take its place. But the new journal will occupy even broader ground than did that, and, we doubt not, will soon become familiar to medical literature. It will be published monthly, simultaneously in the United States and Great Britain, and will be edited by Lewis S. Pilcher, A. M., M. D., of Brooklyn, formerly editor-in-chief of the *Annals of Anatomy*, and C. B. Keetly, F. R. C. S., of London, England, with a very able corps of collaborators in both countries. It will be the only journal in the English language devoted exclusively to surgery. Five dollars per year. Address the publishers.

---

BOSTON, Dec. 16, 1884.

The seventeenth annual meeting of the American Academy of Dental Science was held at Young's Hotel on Wednesday, Nov. 5 1884.

The following officers were elected for the ensuing year:

President, Dr. Geo. T. Moffatt.

Vice-President, Dr. J. H. Batchelder.

Recording Secretary, Dr. E. E. Hopkins.

Corresponding Secretary, Dr. E. B. Hitchcock.

Treasurer, Dr. E. H. Smith.

Librarian, Dr. H. C. Meriam.

Executive Committee, Drs. C. P. Wilson, E. C. Briggs, J. S. Mason.

The annual address was delivered by Dr. E. N. Harris, of Boston, a copy being requested for publication.

E. E. HOPKINS, Rec. Sec'y.

---

### NEW YORK ODONTOLOGICAL SOCIETY.

The officers elected for the ensuing year are:

Dr. Wm. Jarvie, Jr., President.

Dr. J. M. Howe, Vice-President.

Dr. E. T. Payne, Recording Secretary.

Dr. S. E. Davenport, Corresponding Secretary.

Dr. J. S. Goodwillie, Librarian.

Dr. W. A. Bronson, Curator.

Drs. C. A. Woodward, S. G. Perry, H. G. Mirick, Executive Committee.

### THE PENNSYLVANIA SOCIETY REPORT.

In this number we bring to a close the admirable report of the annual meeting of the Pennsylvania State Dental Society, prepared for this journal by Dr. William H. Trueman. It is not every one who can seize upon and present the interesting portion of the debates in such a meeting without loading up the report with that which is of no moment, or of interest to but a small number. Dr. Trueman wrote up the meeting of last year for this journal, and when we had secured his consent to do the same for the sessions of 1884, we were satisfied that the matter was in competent hands, and we are sure that our readers will now agree with us.

---

### DR. SWASEY'S ECONOMIC DISK MOULD.

For years we have been paying twenty-five cents or more apiece for disks that need not cost a tenth of that sum. Dr. James A. Swasey, of Chicago, has devised an apparatus by the use of which corundum and rubber disks may be made of different styles and forms, at an expenditure of very little time and less money. Any dentist or dental assistant can turn them out by the dozen, ready mounted and centered. The apparatus is very simple, as are all really effective utensils.

---

### REMITTANCES.

Some of our subscribers delay paying their subscription fees because they dislike to make out checks for such a small amount, nor do they like to send money through the mails.

We would suggest to our friends that *postal notes* can easily be procured from postmasters, and are a safe and convenient method of remitting their dues.

---

### "DONALDSON'S NERVE BRISTLES."

Just the nicest instruments we have found for removing the contents of pulp canals. Exceedingly delicate and pliable, but firm, tough, and not easily broken. Can be procured at the depots.

C. E. F.

---

### WANTED.

A second-hand Wilkerson chair in good working order. Answer, with price, and where the chair can be seen.

Lock Box 35, Middletown, N. Y.

(37.) In order to correct a mal-articulation for a lad sixteen years of age, whose superior incisors passed behind their inferior neighbors in their occlusion, a metallic plate was made to cover the lower front teeth, to which was soldered an inclined plane attachment. When this was inserted the youth was directed to "bite hard, and bite continually," and the instructions were carried out as faithfully as possible. In three months the upper teeth were brought in proper position, but the severe pressure destroyed the vitality of the two inferior lateral incisors, which are much discolored. It is over fifteen years since the appliance was worn, and the patient observed the change of color about three years later.

As these teeth have never given any annoyance whatever, would it not be well to leave them unmolested, instead of risking disturbance in an attempt to bleach them? R. H. B.

(38.) I have a lady patient who suffers from frequent attacks of acute neuralgic pain, more particularly in the left side of her head and face. Her hair has prematurely turned gray, and her left ear has recently lost its hearing, which her physician (an eminent aurist) pronounces a catarrhal lesion. Quite a number of her teeth, although free from caries, are loose, especially the left superior molars and bicuspids, and at times they are the cause of much pericemental irritation. They are somewhat benefited by treatment, but when treatment is suspended they again become troublesome. It seems hardly possible that the teeth have occasioned the neuralgia, yet their presence may aggravate it. Would it be advisable to extract the teeth, and, if so, could we hope for favorable results? BOSTON.

(39.) What is a fellow to do in such a case as this, and how can he possibly diagnose in advance the true condition of affairs?

Miss H. visited me six months ago, and presented a tooth with an exposure of the pulp. It was in due time capped, and the cavity filled with oxy-phosphate of zinc. There was no pain or soreness following, but the tooth seemed somewhat loose, and kept slowly getting more so. When she came the other day there was entire insensibility to thermal changes, and the tooth seemed devoid of sensation. So I concluded that I had a dead pulp, as I had often had before after such capping, especially as I thought that the color had changed somewhat. I drilled the tooth open without causing any pain, burred out the pulp chamber and thrust a Donaldson broach into the c nal. There was very little sensation, but blood followed the broach, and continued to flow for some time. I don't know what it means. I sealed the cavity up temporarily, and thus it remains, with no pain or soreness. I wish that some one who knows more than I do would tell me what to do with it. CINCINNATUS.

(40.) Why will dentists persist in calling the contents of the pulp canal "the nerve?" If they know anything they must know that the nerve proper forms but a very small proportion of the tissue of the tooth pulp. It sounds ridiculous to hear intelligent dentists speak about the "bleeding of the nerve."

PENNSYLVANIAN.

### Answers.

L. R. (23.) In most cases there is little necessity for cutting the gum around teeth that are to be extracted, especially if there be teeth on each side. But sometimes a tooth stands alone, and there is evidence that the gum is closely adherent, it appearing to be entirely healthy and growing well up on the tooth. This is often the case with wisdom teeth. In such cases it is prudent to press a gum lancet down to the alveolar border when there appears to be adhesion. If there be much inflammation, and especially if there be suppuration or symptoms of pyorrhœa, it is but unnecessary cruelty to cut the gum. I have seen very severe lacerations caused by carelessness or inattention to the condition of the gums previous to extraction, however. C. E. R., NEW JERSEY.

